# Fructus Arctii Mitigates Depressive Disorder via the Let‐7e‐Modulated Toll‐Like Receptor (TLR) Signaling Pathway

**DOI:** 10.1002/brb3.70132

**Published:** 2024-11-13

**Authors:** Weifang Zhang, Qin Zhou

**Affiliations:** ^1^ Department of Anesthesiology Affiliated Hospital of Nantong University Nantong Jiangsu China; ^2^ Department of Pediatric Psychiatry The Affiliated Xuzhou Eastern Hospital of Xuzhou Medical University/Xuzhou Eastern People's Hospital Xuzhou Jiangsu China

**Keywords:** depressive disorder, Fructus arctii, let‐7e, TLR4

## Abstract

**Background:**

Depressive disorder is a common and serious public health challenge globally. Fructus arctii is a traditional medicinal plant ingredient with diverse pharmacological effects. This study aimed to investigate the therapeutic potential of Fructus arctii in alleviating depressive‐like behaviors.

**Materials and Methods:**

We established a chronic unpredictable mild stress (CUMS)‐induced depression mouse model to assess the antidepressant effects of Fructus arctii. BV2 cells treated with lipopolysaccharide (LPS) were used to mimic neuronal damage. Behavioral tests, including the sucrose preference test, tail‐suspension test, and forced swim test, were conducted to evaluate the impact of Fructus arctii on depressive‐like behaviors. Let‐7e expression was detected by RT‐qPCR, and TLR4 signaling pathway activation was evaluated by western blot analysis, which also assessed the inflammatory response by measuring levels of IL‐6, IL‐1β, MCP‐1, TNF‐α, and iNOS. Immunohistological analysis was conducted to detect the expression of microglia markers. Luciferase reporter assays verified the interaction between let‐7e and TLR4.

**Results:**

Fructus arctii administration effectively alleviated depressive‐like behaviors induced by CUMS in mice, as evidenced by improved sucrose preference and reduced immobility time in behavioral tests. Mechanistically, Fructus arctii reversed the CUMS‐induced downregulation of let‐7e and upregulation of TLR4 and MyD88 protein levels in mice hippocampus tissues. In addition, Fructus arctii suppressed microglial activation and reduced the levels of inflammatory factors by upregulating let‐7e. Let‐7e was verified to bind to TLR4, thereby negatively regulating its expression. TLR4 overexpression reversed the suppressive effect of let‐7e upregulation on inflammatory reactions and microglial activation. Furthermore, intracerebroventricular injection of let‐7e agomiR alleviated depressive‐like behavior and inhibited microglial activation in vivo.

**Conclusion:**

In summary, Fructus arctii mitigates depression by regulating the let‐7e/TLR4/MyD88 pathway, offering new insights into potential depression therapies.

## Graphical abstract

Fructus arctii alleviates depressive‐like behaviors by upregulating let‐7e, which inhibits the TLR4/MyD88 signaling pathway, reducing inflammation and microglial activation in response to LPS.

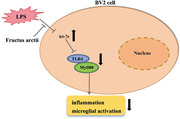

## 1 Introduction

Depressive disorder represents a prevalent psychiatric illness, affecting approximately 11% of individuals worldwide (Mccarron et al. 2021). Characterized by chronic stress, depression exhibits clinical manifestations like persistent sadness, cognitive impairment, sleep disturbance, metabolic irregularity, and suicidal tendency (Dean and Keshavan 2017; Nuñez et al. 2022; Gotlib and Joormann 2010). Risk factors related to depression include gender, age, heightened susceptibility to life stress, and neurological impairments (Dubovsky et al. 2021; J. Li et al. 2015). As a leading cause of disability globally, depression seriously affects the quality of life of patients (Arnow et al. 2015; Rehm and Shield 2019). Current therapeutic approaches mainly include antidepressants and psychotherapies (Myles, Kulkarni, and Nagele 2023). Unfortunately, approximately 25% of patients do not respond to treatment (Lundberg et al. 2023). It is essential to deepen the understanding of the underlying mechanisms of depression for identifying effective treatment targets and developing innovative therapeutic interventions.

MicroRNAs (miRNAs) are a class of small endogenous non‐coding RNAs post‐transcriptionally modulating gene expression by binding to the 3′UTR of target mRNAs (Lu and Rothenberg 2018). Dysregulated miRNAs are implicated in various nervous system disorders, including depression, potentially by regulating the downstream targets and related pathways in depressive disorders (Barbato 2022; Bushati and Cohen 2007; Roy and Dwivedi 2023; Kaurani 2024; Figueroa‐Hall, Paulus, and Savitz 2020). For example, miR‐211‐5p facilitates neurogenesis and reduces neuronal apoptosis, mitigating depression‐like behaviors of depression rats by targeting the Dyrk1A/ASK1/JNK pathway (Shen et al. 2021), whereas miR‐96 exacerbates depressive symptoms through targeting SV2C (Sun et al. 2020). Furthermore, miR‐497 enhances microglial activation and inflammatory responses, exacerbating the depressive process in mice by silencing FGF2 (Zhai et al. 2020). Overall, miRNAs play a pivotal role in modulating gene expression and are implicated in the pathogenesis of depression.

The let‐7 miRNA family, known for its anti‐inflammatory roles, regulates inflammatory factors in various diseases (Kumar et al. 2011; Bernstein, Jiang, and Rom 2021; Lin et al. 2017). Let‐7e, a member of this family, modulates endothelial function and inflammatory responses (Lin et al. 2017). Reduced let‐7e levels have been observed in major depressive disorder patients, correlating with disease severity and increased post‐antidepressant treatment (Hung et al. 2019). Let‐7e is also found downregulated in serum exosome isolated from major depressive disorder patients and is restored post‐antidepressant treatment (Hung et al. 2021). Nonetheless, the underlying mechanisms linking let‐7e to depression remain largely unexplored.

Fructus arctii, derived from *Arctium lappa*, is a traditional medicinal plant rich in active compounds like arctiin and arctigenin, which are known for their antioxidant and anti‐inflammatory properties (Y. Li et al. 2022; J. Hu et al. 2019; Y. Wang et al. 2023). Studies have shown that Fructus arctii can alleviate inflammatory conditions such as sore throat, rashes, asthma, diabetes, and cancers (Y. Li et al. 2022; B. He et al. 2019; Gao, Yang, and Zuo 2018). Arctigenin, in particular, has been demonstrated to alleviate depression by suppressing neuroinflammation via the HMGB1/toll‐like receptor 4 (TLR4)/NF‐κB pathway (Xu et al. 2020). Despite these findings, the therapeutic potential of Fructus arctii in depression warrants further investigation.

In this study, we aimed to elucidate the function and mechanism of Fructus arctii in depression, and we hypothesized that Fructus arctii modulated let‐7e expression to affect depression, which might offer a novel therapeutic target for depression.

## 2 Materials and Methods

### 2.1 Preparation of Fructus Arctii Extract

Dried and ground Fructus arctii powder was obtained from a local market in Nantong, China. Water extract was prepared following the method as described previously (Han et al. 2016). Briefly, the powder was suspended in distilled water at a concentration of 100 g/L and heated to 100°C for 3 h to extract the water‐soluble components. The resulting filtrate was then lyophilized to obtain a dried extract. This dried powder was reconstituted in distilled water to create a stock solution for cellular treatments.

### 2.2 Animal Experiments

Only male C57BL/6J mice aged 6–8 weeks due to the gender differences in response to chronic mild stress (Xing et al. 2013). Mice were purchased from the Affiliated Hospital of Nantong University and housed under a 12‐h light‐dark cycle and constant temperature. All procedures were approved by the Institutional Animal Care and Use Committee (approval no: NU‐277HJAC). Mice were randomly divided into four groups: control (*n* = 12), chronic unpredictable mild stress (CUMS) (*n *= 12), Fructus arctii treatment (*n* = 6), and let‐7e overexpression (*n* = 6). The CUMS protocol involved administering one or two stressors every day, including water and food deprivation (24 h), forced swimming in cold water (40°C, 5 min), white noise (9 h), cage tilting (45°C, 12 h), soiled cage bedding overnight, alteration of the day/night light cycle, and physical restraint (5 min) for 42 days (Jiang et al. 2018; Abuelezz, Hendawy, and Magdy 2017). No single stressor was applied for 3 consecutive days. After 21 days of stress exposure, the Fructus arctii group received 100 mg/kg/day of Fructus arctii extract, whereas the let‐7e group underwent intracerebroventricular injection of let‐7e agomiR or negative control (NC agomiR). Following a 7‐day recovery, mice were exposed to the CUMS protocol mentioned above until Day 42 and subsequently underwent behavioral tests. To assess neuronal morphology, computer‐based cell tracing software Neurolucida 360 (MBF Bioscience, Williston, VT, USA) and Sholl analysis were used following the manufacturer's protocol.

### 2.3 Sucrose Preference Test (SPT)

SPT was conducted following established protocol (Zhao et al. 2021). Briefly, mice were acclimated to drinking water from two bottles equipped with ball‐point sippers over 2 days. On the test day, one bottle contained a 1% sucrose solution, and weight of both bottles was recorded. After 12 h, the bottle positions were swapped to prevent the impact of side preference. The bottles were weighed again at the 24‐h mark. Sucrose preference was determined as the percentage of sucrose solution intake relative to total liquid consumption ([sucrose solution consumed/total liquid consumed] × 100%).

### 2.4 Tail‐Suspension Test (TST)

The TST was conducted following the procedure described in a previous study (Yin et al. 2011). The mouse tail was secured with medical tape onto a plastic rod, with mouse head approximately 50 cm above the testing apparatus's base. Immobility was defined as the absence of any movement. The duration of immobility during the 6‐min test was recorded, and data in initial 2 min for adaptation were excluded, whereas data of the subsequent 4 min were used for analysis.

### 2.5 Forced Swim Test (FST)

The FST is a widely used method to assess depressive‐like behaviors in rodents (Yankelevitch‐Yahav et al. 2011). Mice were introduced into a clear cylindrical tank at 25 cm in diameter, filled with warm water at a depth of 20 cm. After acclimation for 3 min, the immobility time of each mouse over a 4‐min interval was recorded. Immobility was defined as the time during which the mouse floated without making any active movements, ensuring its head remained above the water surface.

### 2.6 Immunohistochemistry (IHC)

The hippocampal tissues were fixed in 4% paraformaldehyde (PFA) for 48 h, dehydrated, and embedded in paraffin. Tissues were cut into 4‐µm thick sections using a microtome and subsequently deparaffinized and rehydrated. For antigen retrieval, sections were treated with citric acid antigen retrieval buffer and heated in a microwave oven. Next, sections were incubated overnight at 4°C with primary antibody (IBA‐1, cell signaling technology #17198, 1:500), followed by incubation for 2 h with the secondary antibody (Abcam). The 3,3′‐Diaminobenzidine (DAB) was used as the chromogen for visualization. After counterstaining with hematoxylin, the sections were dehydrated, mounted, and cover slipped. Observation and analysis were conducted using a light microscope (Olympus).

### 2.7 Cell Culture

BV2 microglial cells, obtained from the Chinese Academy of Medical Sciences in Beijing, were maintained in 96‐well plates (4 × 10^4^ cells/well) in Dulbecco's Modified Eagle Medium (DMEM, Gibco, USA) supplemented with 10% fetal bovine serum (FBS, Gibco, USA) at 37°C in a humidified atmosphere containing 5% CO_2_. Cells were passaged a total of two times every 2–3 days (1:3) using 0.25% trypsin‐EDTA (Gibco, USA) to maintain optimal growth conditions.

### 2.8 Cell Transfection

For let‐7e overexpression or silencing, cells were transfected with either let‐7e mimics, inhibitors, or their respective negative controls (NC) obtained from RiboBio (Guangzhou, China). The sequences used were as follows: Let‐7e Mimic Sequence: 5′‐UGAGGUAGUAGGUUGUAUAGUU‐3′ and Let‐7e Inhibitor Sequence: 5′‐AACUAUACAACCUACUACCUCA‐3′. Transfections were carried out using Lipofectamine 3000 reagent (Invitrogen, USA). Upon reaching 70%–80% confluency, cells were incubated with the transfection complexes for 48 h to facilitate efficient uptake and subsequent modulation of let‐7e expression levels in the cells.

### 2.9 Cell Viability

The effects of Fructus Arctii treatment and lipopolysaccharide (LPS) on the viability of BV2 cells were evaluated using MTT assays. Briefly, cells were exposed to varying concentrations of Fructus Arctii extract (0, 1, 10, 50, 100, 150 µg/mL) or LPS (0, 1, 10, 100, 1000 ng/mL) for 24 h. Following this, 10 µL of MTT reagent (Sigma‐Aldrich, USA) was added to each well and incubated for an additional 4 h at 37°C. The optical density (OD) value was then measured at 570 nm using a microplate reader (Bio‐Rad, Hercules, CA, USA). LPS at 1 µg/mL and Fructus Arctii extract at 100 µg/mL exhibited no cytotoxicity to BV2 cells and were selected for further analyses.

### 2.10 Western Blot

Hippocampal tissues were homogenized in lysis buffer and incubated on ice for 30 min. Subsequently, the homogenates were centrifuged at 12,000 rpm for 15 min at 4°C. The supernatants were then loaded onto a 4%–20% SDS‐PAGE gel for electrophoretic separation, followed by transfer to PVDF membranes. The membranes were blocked with 5% non‐fat milk for 2 h and then incubated overnight at 4°C with primary antibodies against TLR4 (Abcam ab#13556, 1:800), MyD88 (Santa Cruz, #sc‐136970, 1:50), β‐actin (Cell Signaling, #4967, 1:200), IL‐6 (Santa Cruz, #sc‐130326, 1:50), IL‐1β (Abcam, ab#6672, 1:1000), MCP‐1 (Cell Signaling, #2027, 1:200), TNF‐α (Novus Biologicals, #NBP1‐19532, 1:300), and iNOS (Cell Signaling, #2982, 1:400). After washing with TBST, the membranes were incubated with HRP‐conjugated secondary antibodies (Rabbit, ThermoFisher#31460, 1:500; Mouse, ThermoFisher#A‐31430, 1:300) for 1.5 h. Protein bands were visualized using a gel imaging system (Bio‐Rad, Hercules, CA, USA) with an ECL detection kit (Millipore) and analyzed using ImageJ software.

### 2.11 RT‐qPCR

Total RNA was extracted from hippocampal tissues using TRIzol reagent (Thermo Fisher Scientific, Waltham, MA, USA) following the manufacturer's instructions. Subsequently, reverse transcription was performed to synthesize cDNA using a PrimeScript First Strand cDNA Synthesis Kit (Takara, Japan). The quality and concentration of the synthesized cDNA were assessed using a NanoDrop spectrophotometer. Quantitative PCR (qPCR) was conducted using the SYBR Green PCR Master Mix (Takara) on an ABI7500 real‐time PCR system (Applied Biosystems, USA). The relative expression levels of target genes, including let‐7e, TLR4, and MyD88, were normalized to the housekeeping gene β‐actin. Relative gene expression was analyzed using the 2^−ΔΔCt^ method. The sequence of primers used in this study was let‐7e: F: 5′‐UGAGGUAGUAGGUUGUAUAGUU‐3′, R: 5′‐GAUUCUAGUCGAGUCAUGAGCU‐3′ and TLR4 F: 5′‐ GGCTAGGACTCTGATCATGG‐3′ R: 5′‐TTAGGAACTACCTCTATGCAGG‐3′. β‐actin: F: 5′‐GTACCCAGGCATTGCTGACA‐3′, R: 5′‐CGCAGCTCAGTAACAGTCCG‐3′.

### 2.12 Immunofluorescence (IF) Staining

Cells were seeded at a density of 1 × 10^6^ cells per well on glass slides in 12‐well plates and cultured for 48 h. After fixation with 4% PFA for 10 min, cells were permeabilized with 0.1% Triton X‐100 and blocked with 3% bovine serum albumin (BSA, Beyotime, China). Subsequently, cells were incubated overnight at 4°C with primary antibodies against TLR4 (Proteintech, #19811‐1, 1:300) and IBA1 (Proteintech, #81728‐1, 1:200). After washing with PBS, cells were incubated with appropriate fluorophore‐conjugated secondary antibodies (Novus Biologicals, NB7541, 1:400) for 1 h at room temperature. Nuclei were counterstained with DAPI. Fluorescence images were captured using an Olympus fluorescence microscope for analysis.

### 2.13 Fluorescence In Situ Hybridization (FISH)

Specific fluorescence‐conjugated probes for let‐7e and TLR4 were obtained from RiboBio (Guangzhou, China). Paraffin‐embedded tissue sections were dewaxed and rehydrated prior to hybridization and then incubated with the FISH probes in hybridization solution overnight at 37°C. After that, the sections were washed and counterstained with DAPI to visualize nuclei. Fluorescence signals were visualized and captured using an Olympus fluorescence microscope (Olympus, Japan). Probe sequences used in this study were as follows: let‐7e probe: 5′‐Cy3‐GCTCAACTTCACTCCAAG‐3′; TLR4 probe: 5′‐FAM‐AGTTCGAGTCAATAGATCGA‐3′.

### 2.14 RNA Pull‐Down Assay

The biotinylated let‐7e probe sequence (5′‐UGAGGUAGUAGGUUGUAUAGUU‐3′) was custom‐synthesized by RiboBio (Guangzhou, China). BV2 cells were sonicated and lysed to prepare cellular lysates. The lysates were then incubated with streptavidin‐coated magnetic beads (Invitrogen, USA) pre‐bound to the biotinylated let‐7e probe for 2 h at room temperature with gentle agitation. Following incubation, the bead‐probe‐cell lysate mixture was further cultured at 4°C overnight to allow efficient RNA–protein binding. Subsequently, the beads were washed three times with washing buffer to remove unbound proteins. The RNA–protein complexes were then eluted from the beads and purified using standard RNA purification methods and analyzed by RT‐qPCR.

### 2.15 Luciferase Reporter Assay

The pmirGLO luciferase reporter vectors (Promega, USA) were engineered to contain either wild‐type (WT) or mutant (Mut) TLR4 3′UTR sequences, which harbor putative binding sites to let‐7e. The sequences were as follows: WT TLR4 3′UTR: 5′‐AGCUAUGUAGCUAGUCAUGCAU‐3′ and Mut TLR4 3′UTR: 5′‐AGCUAUGUAGUCCGUCAUGCAU‐3′. Cells were co‐transfected with these luciferase reporter constructs along with either let‐7e mimics or negative control (NC mimics) using Lipofectamine 3000 (Invitrogen, USA) for 48 h. After transfection, cells were harvested, and luciferase activity was quantified using the Dual‐Luciferase Reporter Assay System (Promega) to determine the regulatory effect of let‐7e on TLR4 3′UTR.

### 2.16 Bioinformatic Analysis

The UniProt database (https://www.uniprot.org/) collects comprehensive functional annotations for all known protein sequences (Apweiler et al. 2004). From this database, we retrieved the UniProtKB identifiers for TLR4. Subsequently, we imported these identifiers into the Pfam database (http://pfam.xfam.org/) to obtain the Pfam domains associated with TLR4.

### 2.17 Statistical Analysis

Statistical analyses were conducted using GraphPad Prism 8 software. Group differences were assessed by Student's *t*‐test or one‐way ANOVA as appropriate. Data are presented as the mean ± standard deviation (SD) from three independent experiments. *P* value less than 0.05 was considered statistically significant.

## 3 . Results

### 3.1 Fructus Arctii Alleviates Depressive‐Like Behaviors in CUMS Mice

**FIGURE 1:**
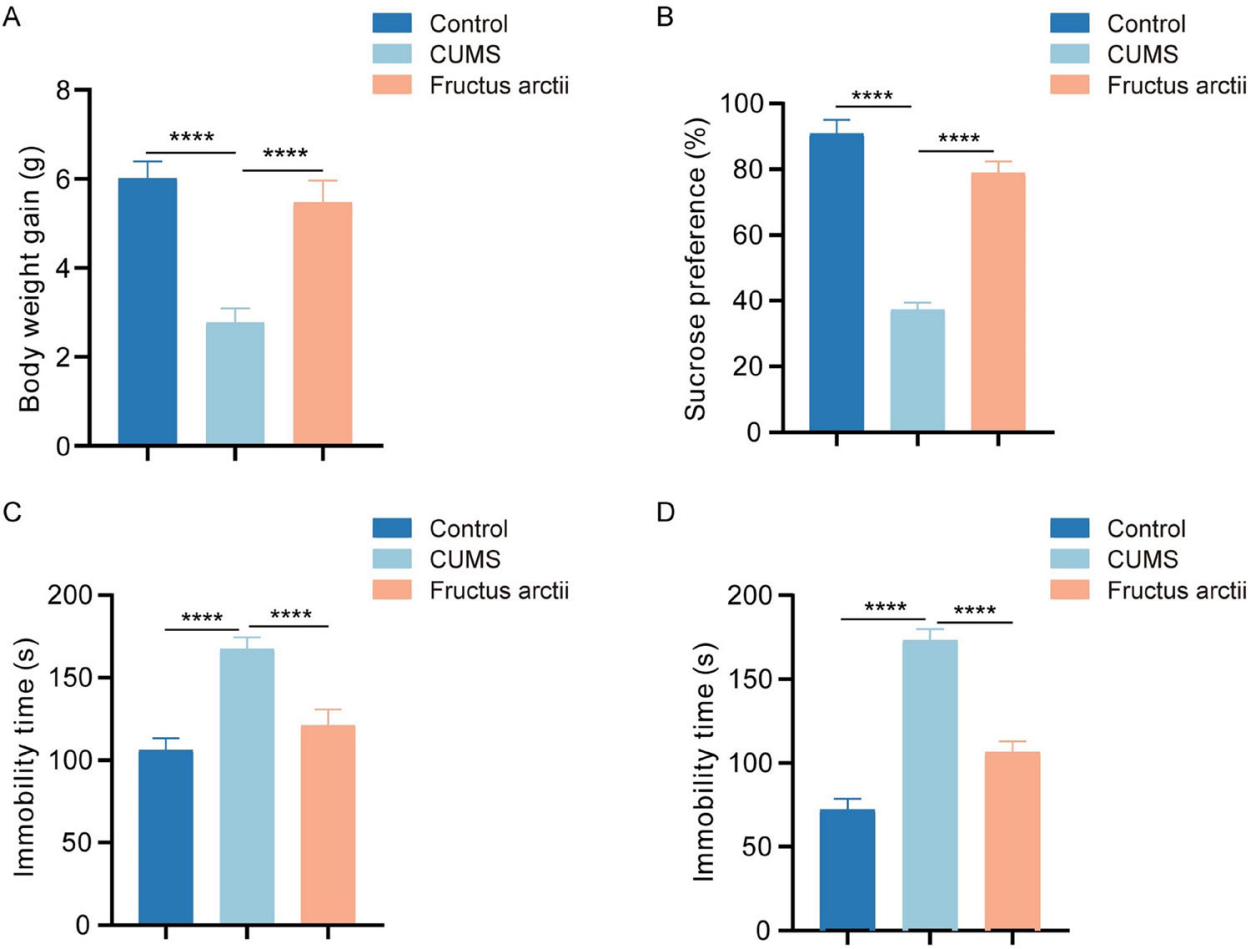
Therapeutic effect of Fructus arctii on depressive‐like behavior in CUMS mice. (A) Changes in body weight in control, CUMS, and Fructus arctii‐treated groups. (B) SPT assessed anhedonia of mice in each group. (C) TST evaluated despair behaviors of mice in each group. (D) FST measured immobility as a sign of depressive‐like behavior in mice in each group. Control and CUMS groups: *n* = 12 mice per group. Fructus arctii: *n* = 6 mice. ****p < 0.0001, one‐way ANOVA.

In our study, we established a CUMS‐induced depression mouse model to evaluate the therapeutic effects of Fructus arctii on depressive‐like behaviors of CUMS mice through multiple behavioral tests. We observed a significant decrease in body weight in CUMS mice compared to controls; however, Fructus arctii administration effectively restored this weight loss (Figure 1A). The SPT used to assess anhedonia—a core symptom of depression—revealed a decline in sucrose preference in CUMS mice. Notably, Fructus arctii treatment reversed this decrease, indicating an improvement in anhedonic behaviors (Figure 1B). Additionally, the TST, commonly used to screen potential antidepressant agents by assessing immobility and behaviors associated with hopelessness, and the FST, another measure of immobility, both demonstrated increased immobility times in mice subjected to CUMS, indicating heightened levels of despair. In contrast, Fructus arctii administration significantly reduced this elevated immobility time, suggesting an antidepressant effect (Figure 1C,D). Overall, our findings demonstrate that Fructus arctii effectively alleviates depressive‐like behaviors induced by CUMS in mice, highlighting its potential as a natural therapeutic agent for depression.

### 3.2 Fructus Arctii Regulates Let‐7e Expression and Suppresses TLR4 Pathway

**FIGURE 2:**
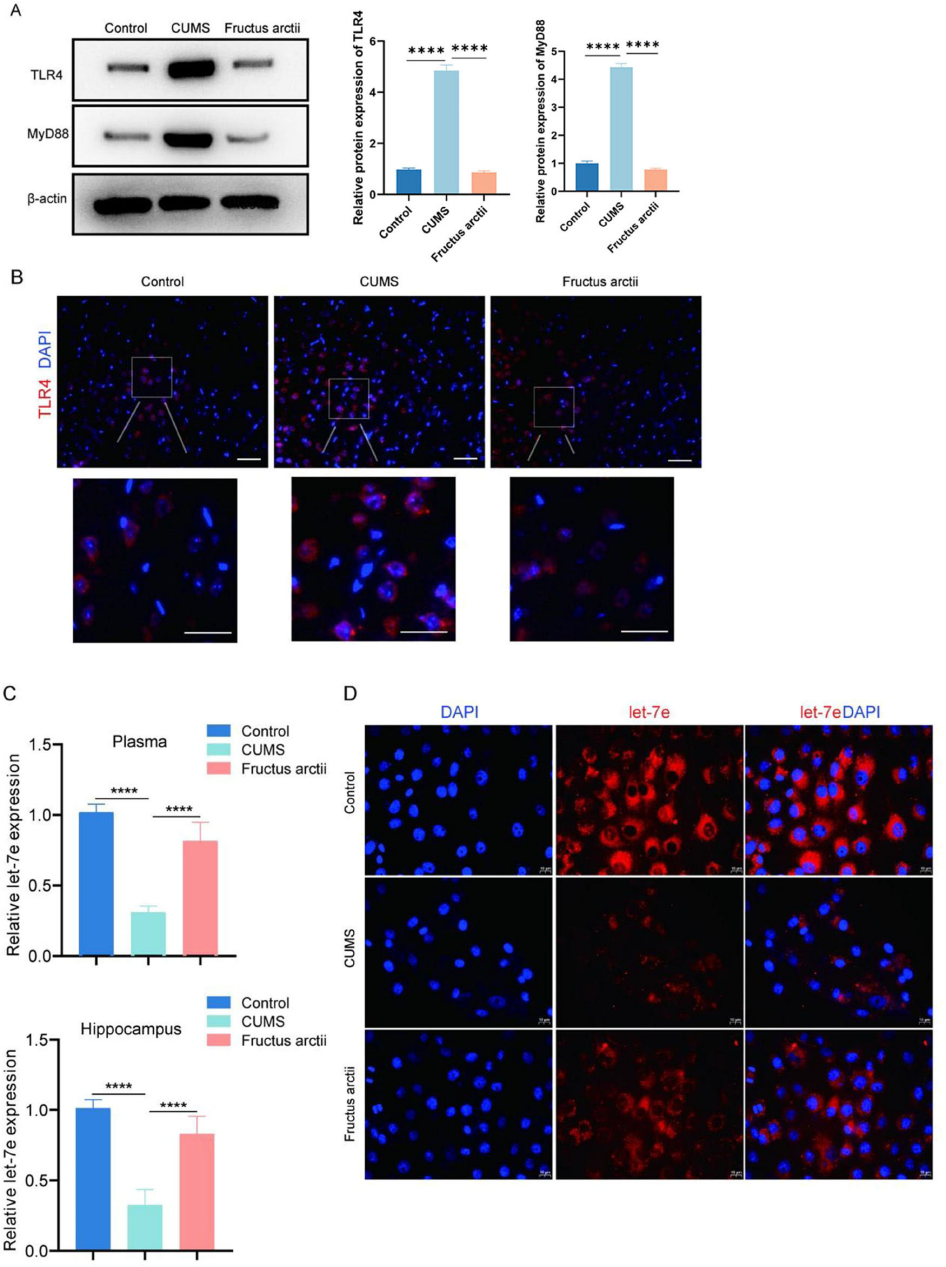
Fructus arctii modulates let‐7e expression and suppresses the TLR4 pathway. (A) Western blot analysis of TLR4 and MyD88 protein levels in the hippocampus of control, CUMS, and Fructus arctii group of mice. Protein expression was normalized to β‐actin. (B) Representative IF images showing TLR4 expression levels in hippocampal sections from different experimental groups. (C) RT‐qPCR analysis depicting let‐7e expression levels in both plasma and hippocampal tissues of the studied mouse groups. (D) FISH assay demonstrating let‐7e expression patterns in mouse hippocampus in each group. Control and CUMS groups: *n* = 12 mice per group. Fructus arctii group: *n* = 6 mice. ****p < 0.0001, one‐way ANOVA.

The TLR4 signaling pathway has been identified as a key player in the inflammatory processes associated with depression (L. Wang and Chen 2017). To investigate the activation of the TLR4 pathway in CUMS depression mice, we conducted a series of experiments. Western blot analysis revealed elevated protein levels of TLR4 and its downstream mediator MyD88 in the hippocampus of CUMS mice compared to controls. Notably, treatment with Fructus arctii significantly reversed these elevated levels (Figure 2A). IF staining further corroborated these findings, demonstrating an increase in TLR4 fluorescence intensity in the hippocampus of CUMS mice. Conversely, administration of Fructus arctii mitigated this heightened fluorescence intensity (Figure 2B). Given the reported involvement of let‐7e in major depressive disorder and its negative regulation of TLR4 expression (Hung et al. 2019; Androulidaki et al. 2009; X. He, Jing, and Cheng 2014), we investigated the impact of Fructus arctii on let‐7e expression levels. RT‐qPCR analysis revealed downregulated let‐7e expression in both plasma and hippocampal tissues of CUMS mice, which was partially restored by Fructus arctii administration (Figure 2C). FISH analysis further confirmed the reduced expression of let‐7e in CUMS mice and the enhancing effect of Fructus arctii on let‐7e expression levels (Figure 2D). Collectively, these results suggest that Fructus arctii upregulates let‐7e expression and inhibits TLR4 pathway activation in CUMS‐induced depression mice.

### 3.3 Fructus Arctii Modulates Microglial Activation via Let‐7e Regulation

**FIGURE 3:**
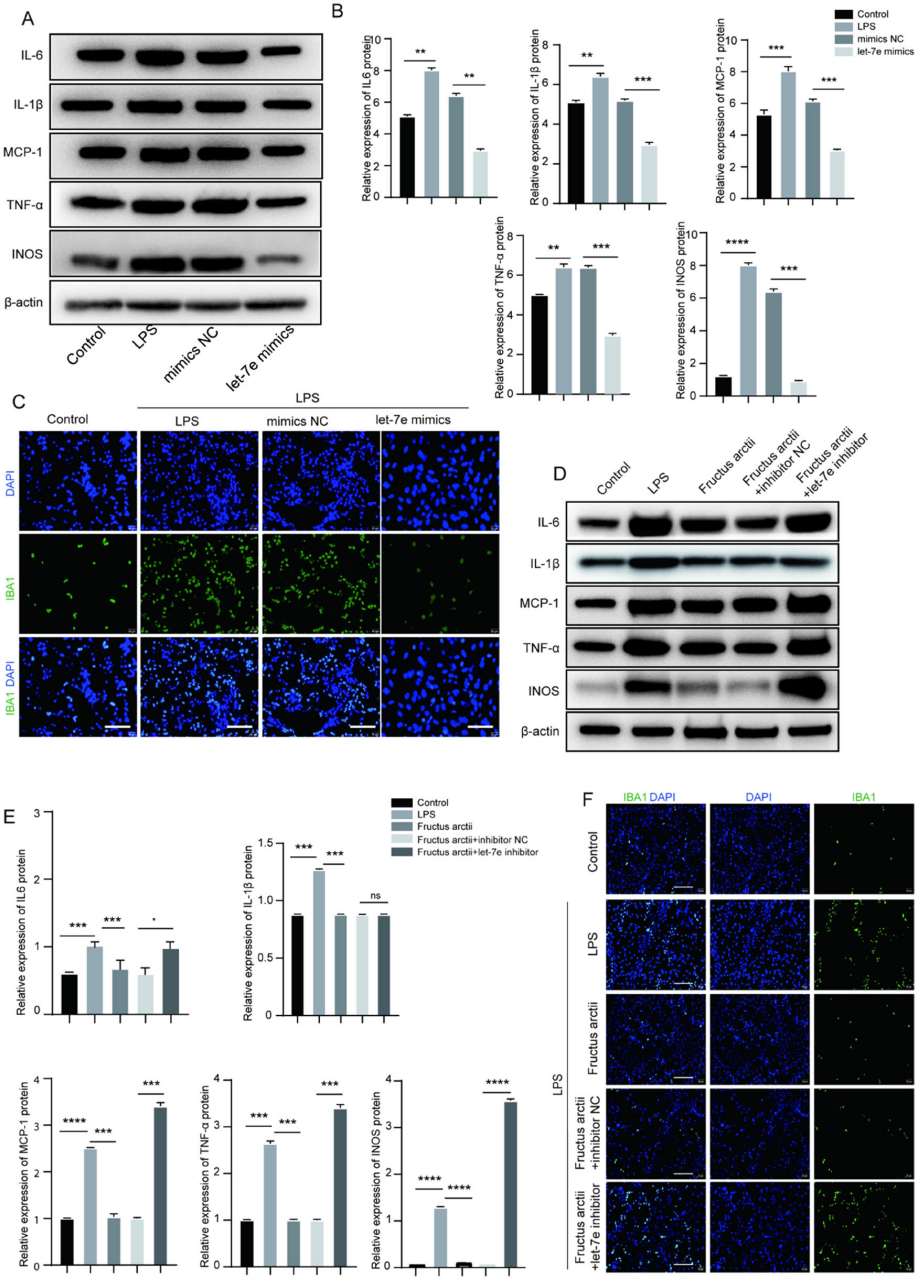
Fructus arctii suppresses activation of microglia via let‐7e. (A, B) Western blot analysis showing the protein levels of IL‐6, IL‐1β, MCP‐1, TNF‐α, and iNOS in BV2 cells treated with LPS alone, LPS + let‐7e mimic, and control cells. (C) Immunofluorescence staining of the microglial marker IBA1 in BV2 cells under different treatments. (D–F) Effect of Fructus arctii and let‐7e knockdown on the expression levels of IL‐6, IL‐1β, MCP‐1, TNF‐α, and iNOS, as well as IBA1 fluorescence intensity. *n* = 3, ns, non‐significant, *p < 0.05, **p < 0.01, ***p < 0.001, ****p < 0.0001, one‐way ANOVA.

To investigate the effects of Fructus arctii on neuroinflammation in vitro, LPS was used to stimulate BV2 cells to induce neuronal damage. The viability of BV2 cells was not significantly affected by LPS at the concentration of 1000 ng/mL (1 µg/mL) (Figure S1A), which was therefore used in subsequent experiments. Let‐7e was overexpressed in LPS‐treated BV2 cells through let‐7e mimic transfection. We then assessed the impact of LPS and let‐7e overexpression on the levels of inflammatory markers. Western blot analysis revealed a significant increase in the protein levels of IL‐6, IL‐1β, MCP‐1, TNF‐α, and iNOS following LPS induction compared to control cells. Interestingly, let‐7e upregulation counteracted the LPS‐induced elevation of these inflammatory markers (Figure 3A,B). IF staining further demonstrated that LPS increased the fluorescence intensity of the microglial marker IBA1, whereas let‐7e overexpression restored microglial morphology to a more normalized state (Figure 3C). Moreover, we investigated the effects of Fructus arctii on LPS‐induced neuroinflammation. MTT assays revealed that Fructus arctii exhibited no toxicity to BV2 cells at concentrations up to 100 µg/mL (Figure S1B). We found that Fructus arctii administration reversed the elevated expression of IL‐6, IL‐1β, MCP‐1, TNF‐α, and iNOS, as well as the increased fluorescence intensity of IBA1 induced by LPS. However, the suppressive effects of Fructus arctii were partially abrogated by let‐7e knockdown with let‐7e inhibitor, resulting in increased inflammatory factor expression and microglial activation (Figure 3D–F). Notably, the impact of let‐7e knockdown on IL‐1β levels was not statistically significant. Collectively, these findings demonstrate that Fructus arctii inhibits microglial activation through the upregulation of let‐7e.

### 3.4 TLR4 Is Targeted by Let‐7e

**FIGURE 4:**
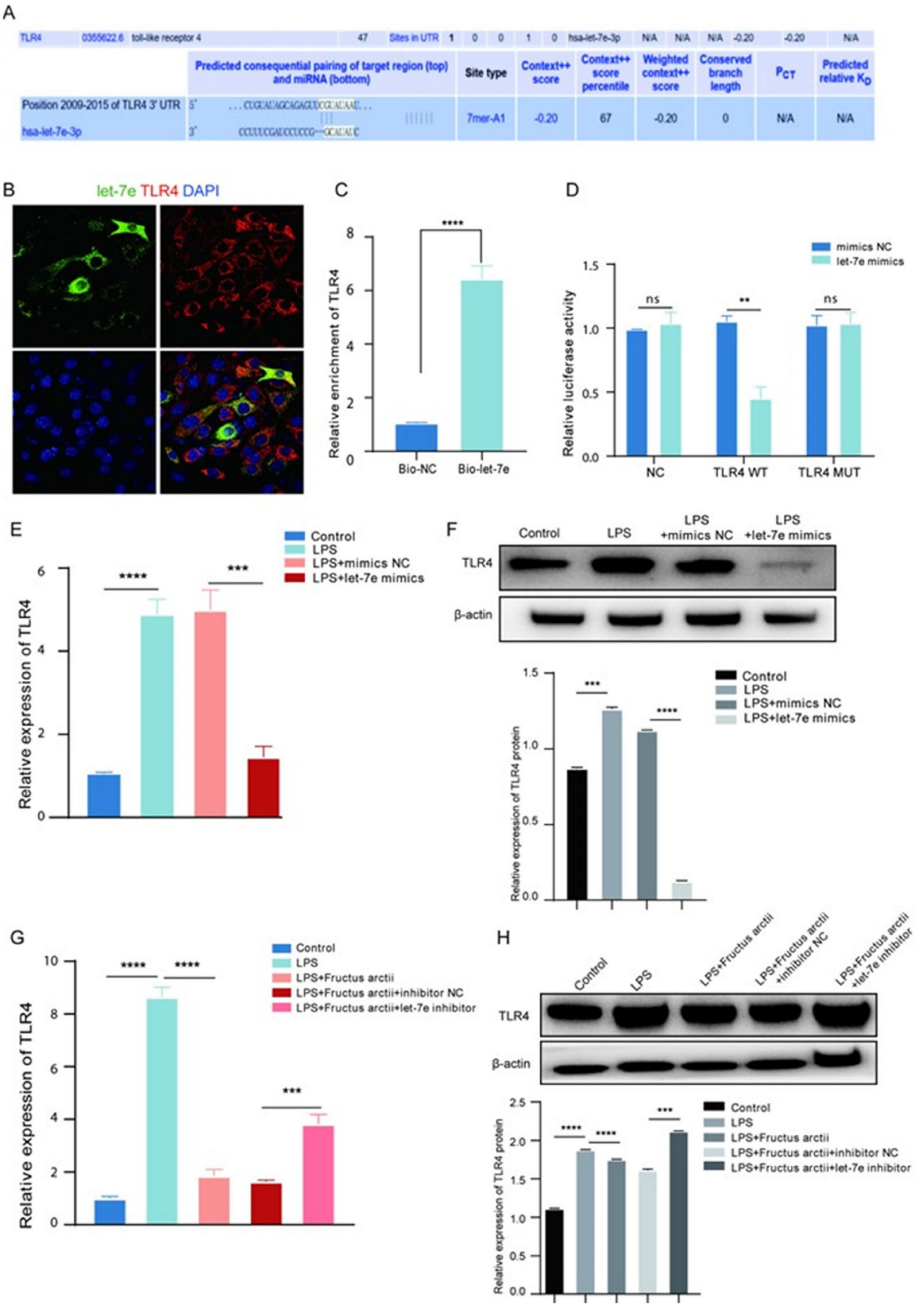
TLR4 is targeted by let‐7e in BV2 cells. (A) Pfam domains associated with TLR4 identified through the UniProt database. (B) FISH analysis revealing co‐localization between let‐7e and TLR4 in BV2 cells. (C) RNA pull‐down assay demonstrating significant enrichment of TLR4 pulled down by the biotinylated let‐7e probe. (D) Luciferase reporter assays indicating reduced TLR4 WT activity upon let‐7e overexpression, with TLR4 MUT activity remaining unchanged. (E) RT‐qPCR analysis of TLR4 mRNA expression in control, LPS, LPS + mimics NC, and LPS + let‐7e mimics‐treated BV2 cells. (F) Western blot analysis showing TLR4 protein levels in each treatment group. (G) RT‐qPCR assessment of TLR4 mRNA expression in control, LPS, LPS + Fructus arctii, LPS + Fructus arctii + inhibitor NC, and LPS + Fructus arctii + let‐7e inhibitor groups. (H) Western blot analysis depicting TLR4 protein levels in each treatment group. *n* = 3; ns, non‐significant; **p < 0.01, ***p < 0.001, ****p < 0.0001; Student's *t*‐test or one‐way ANOVA.

Given the prominent role of TLR4 in mediating pro‐inflammatory responses upon LPS stimulation, we investigated the regulatory relationship between let‐7e and TLR4 in BV2 cells. Based on the UniProt database, we gathered essential information and identified Pfam domains associated with TLR4 (Figure 4A). Through FISH analysis, we observed co‐localization between let‐7e and TLR4 in BV2 cells (Figure 4B). RNA pull‐down assays further corroborated the interaction between let‐7e and TLR4, with the significant enrichment of TLR4 pulled down by biotinylated let‐7e probe (Figure 4C). Subsequently, luciferase reporter assays revealed a marked reduction in TLR4 WT activity after let‐7e overexpression, whereas TLR4 MUT activity remained largely unaffected (Figure 4D), indicating a direct interaction between let‐7e and TLR4. We further assessed TLR4 expression using RT‐qPCR and western blot in control, LPS, LPS + mimics NC, and LPS + let‐7e mimics‐treated BV2 cells. LPS treatment led to elevated TLR4 expression at both mRNA and protein levels, which was subsequently reversed by let‐7e overexpression (Figure 4E,F). Additionally, we also evaluated the impact of Fructus arctii on TLR4 expression in various treatment groups, and Fructus arctii treatment was revealed to mitigate the LPS‐induced upregulation of TLR4, particularly at the mRNA level. However, this suppressive effect was partially reversed by let‐7e inhibition, leading to increased TLR4 expression (Figure 4G,H). Collectively, our findings indicate that TLR4 is a direct target of let‐7e.

### 3.5 Let‐7e Modulates Microglia Activation by Targeting TLR4

**FIGURE 5:**
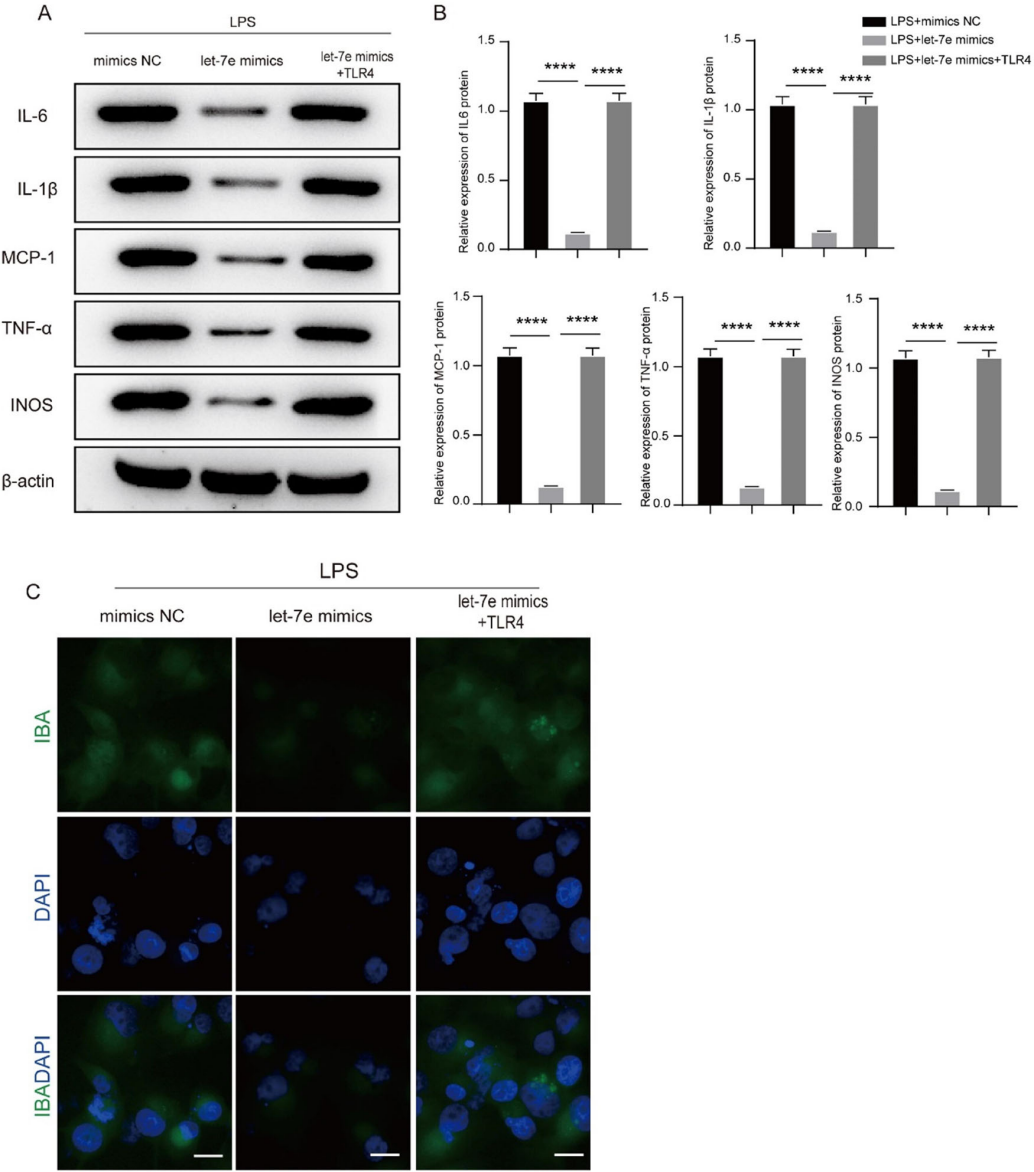
Let‐7e modulates microglia activation by targeting TLR4. (A, B) Western blot analysis of pro‐inflammatory markers (IL‐6, IL‐1β, MCP‐1, TNF‐α, and iNOS) in LPS‐induced BV2 cells with let‐7e overexpression or TLR4 upregulation. (C) Immunofluorescence staining of IBA1 fluorescence intensity in BV2 cells illustrating the effects of let‐7e and TLR4 on microglial activation. *n *= 3, ****p < 0.0001, one‐way ANOVA.

Given the pivotal role of the let‐7e/TLR4 axis in modulating inflammation, we further investigated its impact on microglia activation. TLR4 was overexpressed, and the overexpression efficacy was validated by RT‐qPCR (Figure S2A). Meanwhile, we found that the let‐7e overexpression induced TLR4 downregulation in LPS‐treated BV2 cells was reversed by transfection of TLR4 overexpression vector (Figure S2B). Furthermore, western blot analysis revealed that overexpression of let‐7e in LPS‐induced BV2 cells led to a significant reduction in the protein levels of pro‐inflammatory markers IL‐6, IL‐1β, MCP‐1, TNF‐α, and iNOS, whereas upregulation of TLR4 showed the opposite effect (Figure 5A,B). IF staining corroborated these findings, showing that the reduction of IBA1 fluorescence intensity induced by let‐7e overexpression was reversed upon TLR4 upregulation (Figure 5C). Overall, these results substantiate that let‐7e modulates microglia activation by targeting TLR4.

### 3.6 Let‐7e Alleviates Depressive‐Like Behavior and Suppresses Microglia Activation in CUMS Mice

**FIGURE 6:**
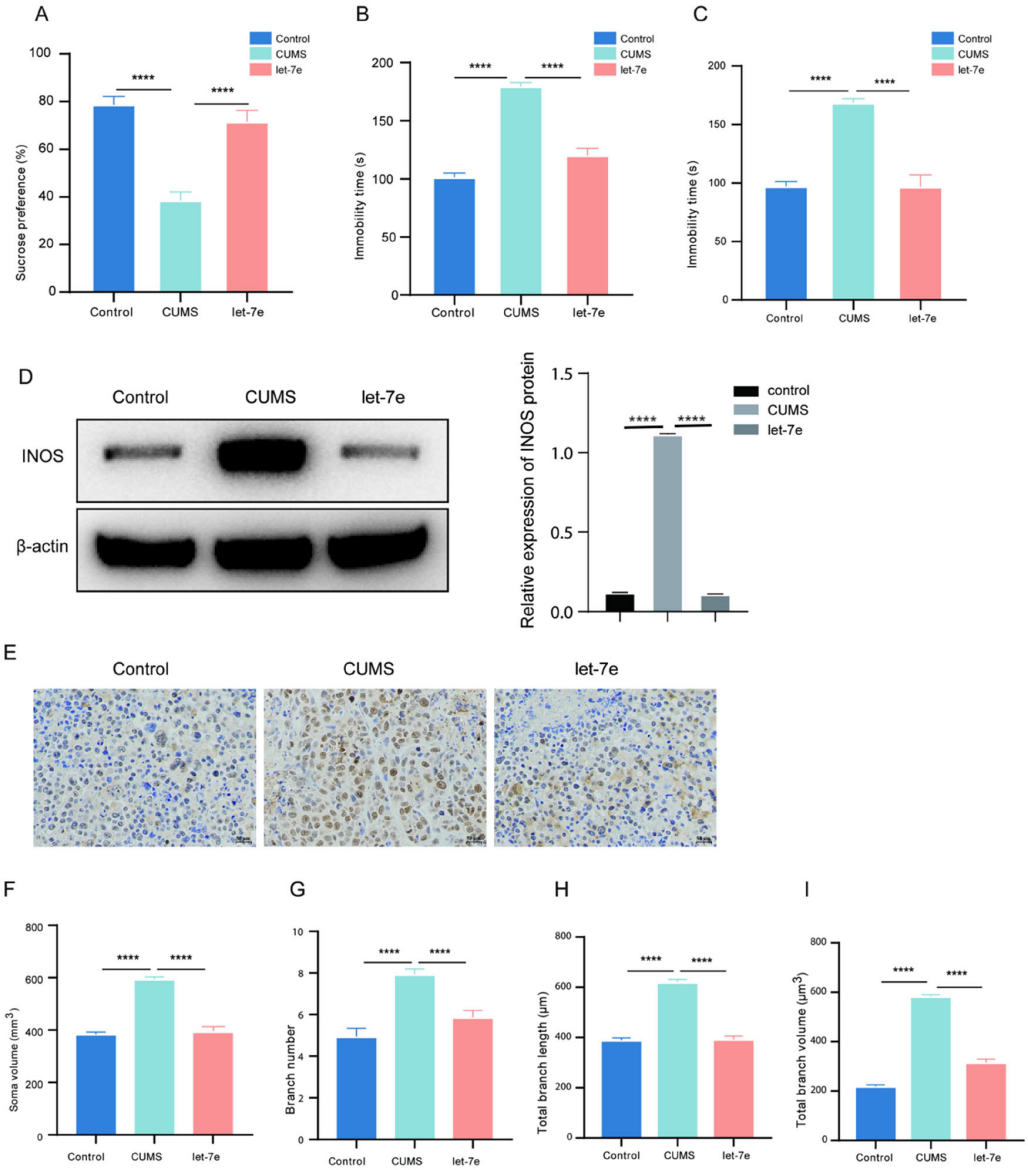
Let‐7e overexpression mitigates depressive‐like behavior and suppresses microglial activation in CUMS mice. (A–C) SPT, TST, and FST tests were conducted to assess depressive‐like behaviors in mice in the control, CUMS, and let‐7e agomiR‐treated groups. (D) Western blot analysis of iNOS protein levels in the hippocampus of control, CUMS, and let‐7e agomiR‐treated CUMS mice. (E) Immunohistochemical staining showing IBA1 levels in the hippocampus of control, CUMS, and let‐7e agomiR‐treated CUMS mice. (F–I) Measurement of microglia showing changes in soma volume, branch number, branch length, and branch volume in each group of mice. Control and CUMS groups: *n* = 12 mice per group. Let‐7e overexpression group: *n* = 6. ****p < 0.0001, one‐way ANOVA.

To investigate the effects of let‐7e on depressive‐like behavior, CUMS mice were administered with let‐7e agomiR. We observed that let‐7e overexpression effectively reversed the CUMS‐induced reduction in sucrose preference rate and increased immobility time, as evidenced by the SPT and FST (Figure 6A–C). Given the known association between elevated iNOS levels and inflammatory responses in depressive states, we measured iNOS protein levels in the hippocampus. Consistent with our hypothesis, let‐7e agomiR administration led to a significant reduction in iNOS protein levels compared to untreated CUMS mice (Figure 6D). Immunohistochemical analysis further revealed that let‐7e overexpression markedly decreased the elevated IBA1 levels in the hippocampus of CUMS mice, indicating reduced microglial activation (Figure 6E). Additionally, analysis of microglia showed that CUMS‐induced changes, including increased soma volume, branch number, branch length, and branch volume, were attenuated by let‐7e overexpression (Figure 6F–I). Collectively, these findings demonstrate that let‐7e plays a pivotal role in alleviating CUMS‐induced depressive‐like behavior and inhibiting microglial activation in mice.

## 4 Discussion

Depression, a chronic psychiatric disorder influenced by psychological, social, and biological factors, remains a significant global health concern (Remes, Mendes, and Templeton 2021). Stress as a primary risk factor contributes to the onset and progression of depression (Gold 2015; Wallensten et al. 2023). Fructus arctii, a traditional medicinal plant, has shown promise for the treatment of various diseases, including sore throat, diabetes, and cancer (Y. Li et al. 2022). Notably, it attenuates diabetic kidney disease by activating protein phosphatase 2 in podocytes (Zhong et al. 2019) and inhibits mast cell‐mediated allergic responses, suggesting its potential in allergic inflammatory diseases (Kee and Hong 2017). In this study, we established a CUMS‐induced mouse model to explore the therapeutic potential and underlying mechanisms of Fructus arctii against depression. Our findings indicate that Fructus arctii effectively mitigates depressive‐like behaviors in CUMS mice, implicating its role in depression management.

miRNAs play pivotal roles as post‐transcriptional regulators, modulating various cellular processes (Diener, Keller, and Meese 2022). Several studies have indicated the association between aberrantly expressed miRNAs and depression pathogenesis (Żurawek and Turecki 2021; Ding et al. 2023). Among these, let‐7e, a member of the let‐7 family, emerges as a critical modulator of inflammatory responses and has been inversely correlated with depression severity (Hung et al. 2019). Our study reveals that Fructus arctii improves behavioral outcomes by regulating let‐7e expression and suppressing TLR4 in CUMS mice, suggesting its therapeutic potential via regulating let‐7e.

Neuroinflammation, marked by microglial activation, is increasingly recognized as a significant contributor to depression pathogenesis (Ignácio et al. 2019; F. Liu et al. 2023; S. Liu, Gao, and Zhou 2022). Enhanced microglial activation has been observed in depression patients, underscoring the importance of neuroinflammation in this disorder (Brites and Fernandes 2015; Fang et al. 2023). Such activation can induce the release of inflammatory factors, impair neural plasticity, and contribute to cognitive dysfunction, exacerbating depressive symptoms (Walker, Nilsson, and Jones 2013; Singhal and Baune 2017; H. Wang et al. 2022). Patients with depression frequently exhibit elevated levels of pro‐inflammatory factors (Haapakoski et al. 2016; Pastis, Santos, and Paruchuri 2023). Consistent with these observations, our study revealed enhanced IBA1 activity and increased expression of pro‐inflammatory cytokines (IL‐6, IL‐1β, MCP‐1, and TNF‐α) and the inflammatory mediator iNOS in LPS‐stimulated BV2 microglia. Notably, both let‐7e overexpression and Fructus arctii administration effectively attenuated these pro‐inflammatory responses induced by LPS. Moreover, the protective effects of Fructus arctii were compromised by let‐7e knockdown, emphasizing the critical role of let‐7e in Fructus arctii‐mediated anti‐inflammatory effects. Although let‐7e has been associated with anti‐inflammatory effects in conditions like allergic rhinitis (L. Li et al. 2018) and tumor suppression through various pathways (G. Li et al. 2024; Niculae et al. 2022; Y. Li et al. 2021), it can also promote inflammation through PI3K/Akt signaling pathway in spinal cord injury mice model (Tang et al. 2019). Inhibition of let‐7e has been shown to attenuate cardioprotection against ischemia‐reperfusion injury through the Akt and mTOR signaling pathways (J. Li et al. 2016). Additionally, let‐7 knockdown has demonstrated neuroprotective effects post‐cerebral ischemia‐reperfusion injury by upregulating mitogen‐activated protein kinase phosphatase 1 (MKP1) expression, leading to reduced apoptosis and inflammation (Z.‐K. Wang et al. 2016). Interestingly, upregulation of miR‐let‐7a in microglia has been associated with promoting anti‐inflammatory factors and safeguarding microglia against apoptotic damage (Cho et al. 2015). In our study, Fructus arctii was found to inhibit microglial activation and reduce neuroinflammation by upregulating let‐7e, suggesting a potential mechanism for its antidepressant effects.

The TLR4/MyD88 signaling pathway is implicated in depression pathophysiology (Shao et al. 2021). TLR4, expressed in brain microglia, plays a crucial role in recognizing LPS and triggering pro‐inflammatory responses via pathways such as NF‐κB and Akt signaling (T.‐Y. Hu et al. 2019; Udomruk et al. 2018). TLR4 activates the MyD88‐dependent pathway, leading to nuclear translocation of NF‐κB and subsequent release of pro‐inflammatory factors (Kawai and Akira 2006; Kim et al. 2023). Notably, CUMS increases TLR4 and MyD88 expression, amplifying NF‐κB activation and inflammatory responses in mouse hippocampi (Yang et al. 2019). TLR4‐deficient mice display no depressive phenotype, cognitive impairments, or neuroinflammation compared to depression‐model mice (Badshah, Ali, and Kim 2016; Cheng et al. 2016). Our study demonstrated that CUMS‐induced elevation in TLR4 and MyD88 expression was restored by Fructus arctii treatment. We also found the co‐localization of TLR4 and let‐7e in cytoplasm of BV2 cells and identified let‐7e as a negative regulator of TLR4. Fructus arctii reduced LPS‐induced TLR4 expression, whereas TLR4 overexpression counteracted the suppressive effects of let‐7e upregulation on microglial activation. Overall, we confirmed that Fructus arctii alleviated depression by modulating the let‐7e/TLR4 axis. The animal experiments further revealed that let‐7e hippocampal injection significantly alleviated depressive‐like behaviors and microglial activation in CUMS mice.

Although our study provides compelling evidence for the therapeutic potential of Fructus arctii in alleviating depressive symptoms through the let‐7e‐mediated TLR4/MyD88 signaling pathway, it still has some limitations. Further research is needed to explore the long‐term effects and safety profile of Fructus arctii in diverse populations, as well as to elucidate the precise mechanisms underlying the interaction between let‐7e and TLR4 in depression. Additionally, only male mice were used in this study, which may limit the generalizability of our findings. Future studies could focus on investigating the therapeutic efficacy of Fructus arctii in combination with standard antidepressant medications to potentially enhance treatment outcomes for depression.

In summary, our findings demonstrate that Fructus arctii mitigates depressive disorder via the let‐7e‐mediated TLR4/MyD88 signaling pathway, suggesting its potential as a therapeutic agent for depression.

## Author Contributions

W.Z. and Q.Z. conceived and designed the study, conducted the experiments, analyzed the data, performed statistical analysis, and contributed to drafting and revising the manuscript.

## Ethics Statement

The experimental protocol was approved by the Institutional Animal Care and Use Committee (approval no: NU‐277HJAC).

## Conflicts of Interest

The authors declare no conflicts of interest.

### Peer Review

The peer review history for this article is available at https://publons.com/publon/10.1002/brb3.70132.

## Supporting information



Figure S1: Cytotoxicity of Fructus Arctii and LPS to BV2 cells.
Figure S2: TLR4 Overexpression Efficacy in BV2 Cells.

## Data Availability

The data that support the findings of this study are available from the corresponding author upon reasonable request.
