# Supplementary material for: Fructus Arctii Mitigates Depressive Disorder via the Let‐7e‐Modulated Toll‐Like Receptor (TLR) Signaling Pathway
Source: Brain Behav. 2024 Nov 13;14(11):e70132. doi: 10.1002/brb3.70132 (PMC11560858; doi:10.1002/brb3.70132)
Supplement: Supplementary file 1 — Figure S1: Cytotoxicity of Fructus Arctii and LPS to BV2 cells. Figure S2: TLR4 Overexpression Efficacy in BV2 Cells. [file BRB3-14-e70132-s001.docx]

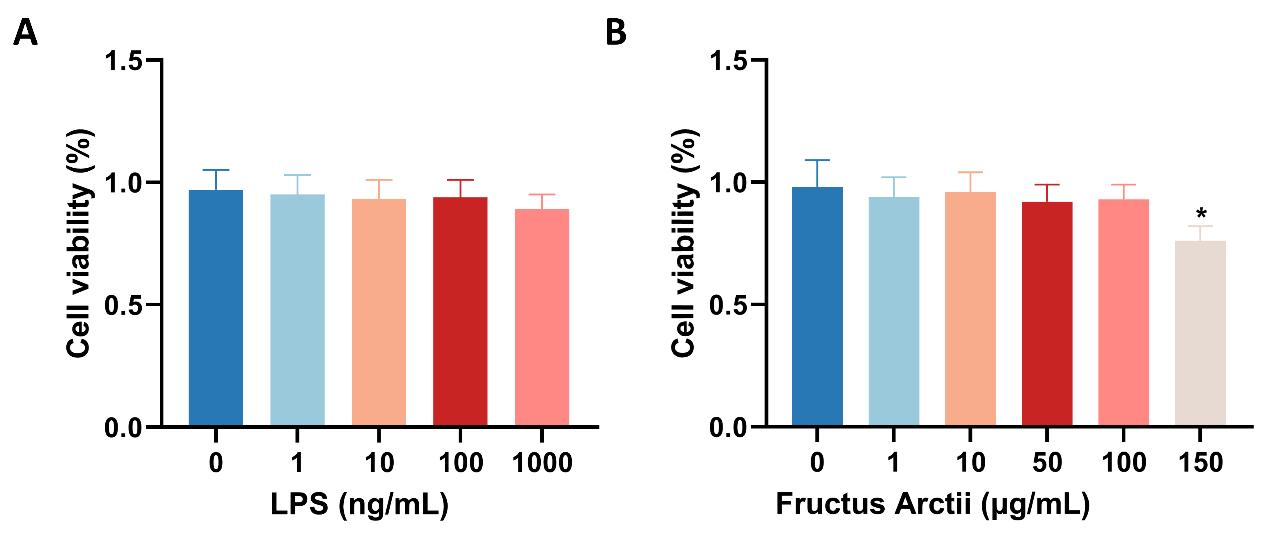


**Figure S1: Cytotoxicity of Fructus Arctii and LPS to BV2 cells.** MTT assays were conducted to assess the viability of BV2 cells treated with various concentrations of (A) LPS or (B) *Fructus arctii* for 24 hours. n=3, *P<0.05, one-way ANOVA.


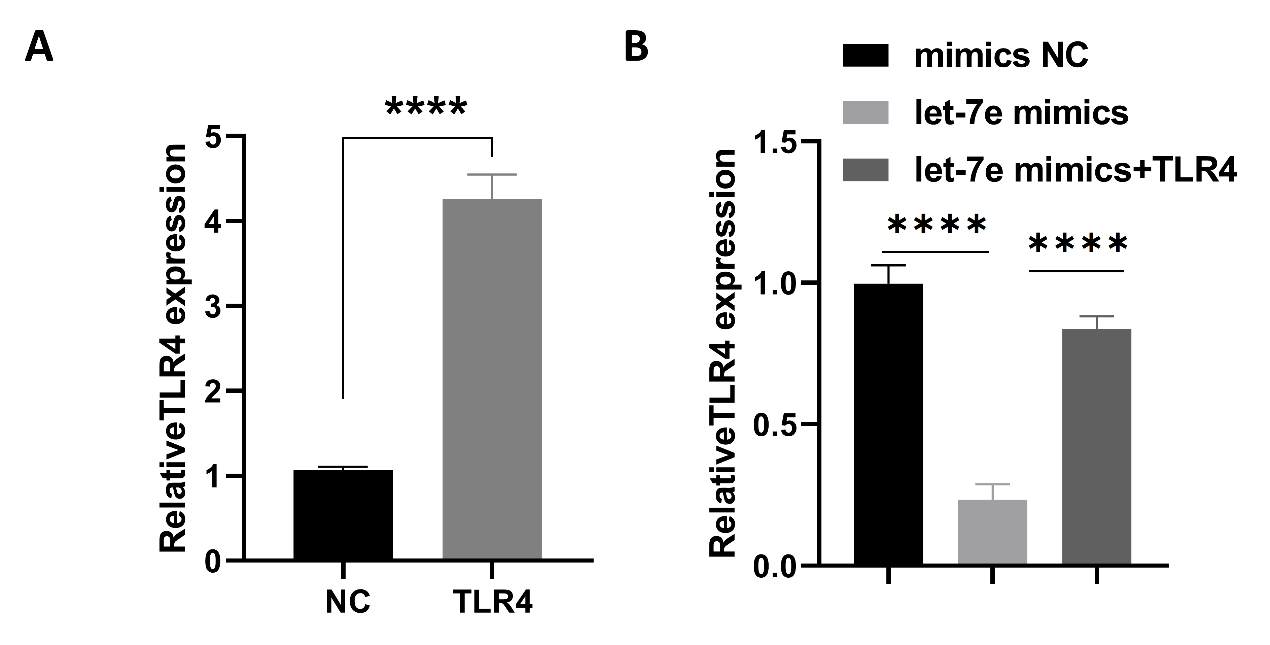


**Figure S2: TLR4 Overexpression Efficacy in BV2 Cells.** (A) RT-qPCR was used to detect the overexpression efficiency of TLR4 in BV2 cells. (B) RT-qPCR was used to detect the TLR4 mRNA expression in LPS-treated BV2 cells with let-7e overexpression or TLR4 upregulation. n=3, ****P<0.0001, Student’s t test or one-way ANOVA.
